# Transcranial direct current stimulation combined with trunk-targeted, proprioceptive neuromuscular facilitation in subacute stroke: a randomized controlled trial

**DOI:** 10.7717/peerj.13329

**Published:** 2022-04-28

**Authors:** Jaya Shanker Tedla, Erika Rodrigues, Arthur S. Ferreira, Jose Vicente, Ravi Shankar Reddy, Kumar Gular, Devika Rani Sangadala, Venkata Nagaraj Kakaraparthi, Faisal Asiri, Ajaya Kumar Midde, Snehil Dixit

**Affiliations:** 1 Department of Medical Rehabilitation Sciences, College of Applied Medical Sciences, King Khalid University, Abha, Aseer, Saudi Arabia; 2 Postgraduate Program in Rehabilitation Science, University Center Augusto Motta UNISUAM, Rio de Janeiro, Brazil; 3 Professor of Neurological Physiotherapy, Federal University of Rio de Janeiro, Rio de Janeiro, Brazil; 4 Head of Neurorehabilitation, Department of Physiotherapy, Krishna Institute of Medical Sciences, Secunderabad, Telangana, India

**Keywords:** Proprioceptive neuromuscular facilitation, Physical therapy, Stroke, Transcranial direct current stimulation

## Abstract

**Background:**

Stroke is the foremost cause of death and disability worldwide. Improving upper extremity function and quality of life are two paramount therapeutic targets during rehabilitation.

**Aim of the study:**

To investigate the effects of transcranial direct current stimulation (tDCS) combined with trunk-targeted proprioceptive neuromuscular facilitation (PNF) on impairments, activity limitations, and participation restrictions of subjects with subacute stroke.

**Methodology:**

Fifty-four subjects with subacute stroke were divided into three groups using block randomization. All three groups received rehabilitation sessions lasting 90 min in duration, four times per week, for 6 weeks. Group 1 (*n* = 18) received conventional physical therapy (CPT); group 2 (*n* = 18) received CPT, trunk-targeted PNF, and sham tDCS; and group 3 (*n* = 18) received CPT, trunk-targeted PNF, and bihemispheric motor cortex stimulation with tDCS. Changes in motor impairment, motor activity, and health-related quality of life assessments were outcome measures.

**Results:**

A two-way linear mixed model analysis revealed interaction effects (group × time) for all outcome measurements (Trunk Impairment Scale, Fugl-Meyer Assessment of Motor Recovery after stroke upper extremity subsection, Wolf Motor Function Test, 10-Meter Walk Test, and the Stroke-Specific Quality of Life scale; all *p* < 0.01 or lower). Overall, post–pre mean differences demonstrate more substantial improvement in the active tDCS group, followed by sham stimulation associated with the PNF group and the group that received CPT alone.

**Conclusion:**

Trunk-targeted PNF combined with bihemispheric tDCS along with CPT engender larger improvements in upper extremity and trunk impairment, upper limb function, gait speed, and quality of life in the subacute stroke population.

## Introduction

Stroke is the foremost cause of death and disability worldwide (Mendis, Davis & Norrving, 2015; Xie et al., 2006). Activities of daily living and quality of life are affected in subjects with stroke (Pohl et al., 2011), and most subjects do not regain complete upper extremity function 6 months post-stroke (Winters et al., 2016). Even with treatment from state-of-the-art facilities, including multidisciplinary teams, the functional improvements in stroke are modest, especially in upper limb recovery (French et al., 2007).

Several studies show an association of trunk control with upper and lower extremities functions, mobility balance, and gait (Isho & Usuda, 2016; Karthikbabu et al., 2018), and trunk control can predict a moderate level of attainable functional recovery in post-stroke subjects (Verheyden et al., 2007; Di Monaco et al., 2010). Trunk control is a vital component to be addressed during the rehabilitation of stroke subjects (Karthikbabu et al., 2012; Lee & Kim, 2018). Furthermore, restraining abnormal compensatory movements and providing trunk stabilization improves upper extremity functions (Wee et al., 2015). In a similar manner, selective trunk exercises designed to improve trunk coordination and control (Karthikbabu et al., 2012) can boost balance and gait parameters among stroke subjects (Li et al., 2017).

Proprioceptive Neuromuscular Facilitation (PNF) is a concept of treatment designed for improving the functional capabilities of people suffering from neurological and traumatic conditions. It is aimed to achieve the highest level of function in the person by using a positive approach. The approach utilizes motor control and motor learning principles and mobilizes the individual potentials with intensive physical training using basic concepts and facilitation techniques in the various positions (Adler, Beckers & Buck, 2014). In the PNF approach, they stimulate proprioceptors in the muscles and joints of the body by using facilitation techniques in the spiral patterns, through which they reorganize the neuromuscular integration and improves the functional capabilities of the patients (Westwater-Wood, Adams & Kerry, 2010). More recently, studies have assessed the effects of trunk-targeted exercises using PNF techniques (Shinde & Ganvir, 2014), which show superior or similar effects in comparison to conventional exercises in improving function, balance, and gait (Chaturvedi, 2017).

Transcranial direct current stimulation (tDCS) is a non-invasive method of brain stimulation with a capability to alter cortical excitability that has gained popularity in recent literature on strokes (Sánchez-Kuhn et al., 2017). tDCS is an easy-to-use, inexpensive, and safe modality of stimulation that can be applied in stroke rehabilitation (Chhatbar et al., 2017). Even though there are randomized controlled trials showing that tDCS is a valuable modality in treating stroke, recent systematic reviews and meta-analysis present contradictory results for the application of this method in stroke rehabilitation (Sánchez-Kuhn et al., 2017; Elsner, Kugler & Mehrholz, 2018; Elsner et al., 2017; Gomez Palacio Schjetnan et al., 2013). Factors such as stimulation parameters, patient characteristics, and time after stroke are some of the elements that can account for the variability in the potential benefits of tDCS (Fuentes Calderón et al., 2019). In addition, the type of adjuvant therapy must be selected carefully to engender further improvements through tDCS (Marquez et al., 2015). Combining the neuromodulation capability of tDCS with a robust clinical treatment tool such as PNF could result in further functional improvements in stroke survivors. Cho & Cha (2014) recently conducted a study utilizing similar approach of combining trunk targeted PNF with tDCS and seen its improvements in lower extremity functions. They proposed future investigation on larger sample with other objective and quantitative outcome measures (Cho & Cha, 2014). Currently, there is a dearth of literature pertaining to this combination of tDCS with PNF in improving upper limb function and quality of life in subjects with subacute stroke. This study aims to assess the effects of tDCS combined with trunk targeted PNF on impairments, activity limitations, and participation restrictions of individuals with subacute stroke.

## Methods

### Ethics and study design

**Figure 1:**
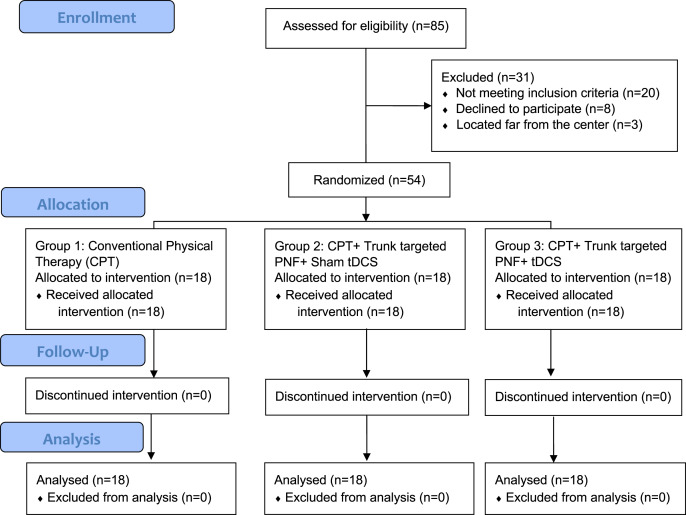
Showing enrollment, allocation, follow-up, and analysis of number subjects.

This is a double-blind, randomized controlled trial; both patients and assessors were blinded to the group allocation. The total duration of the study was 1 year and it was conducted between 2019 and 2020 in a tertiary care hospital setup. We followed CONSORT guidelines, the study was approved by the institutional ethical committee (ECM#2019-31) of the Deanship of Scientific Research, King Khalid University and a clinical trial was registered (CTRI/2019/09/021270). We used convenient sampling to recruit participants and all participants were informed about the study process and signed the written informed consent to participate. By using G* Power statistical software version 3.1.9.5 we estimated the sample size: The two tailed alpha error was at 5%, with a power of the study at 95%, effect size of 0.5 along with including 20% dropout rate the total sample needed for the study was 54. Details of patient recruitment and other processes of allocation, follow-up, and analysis are shown in Fig. 1. Fifty-four patients were block randomized with concealed allocation into three groups (*n* = 18 patients each) by opaque envelopes with each block size of nine patients and the Reddy RS was responsible for blinding. All patients were recruited from our hospital after obtaining the written informed consent of participation.

### Participants

Male and female individuals with stroke were included in the study after fulfilling the following criteria: age >18 years, first-time supratentorial stroke diagnosed by MRI/CT scan, onset of stroke between 7 days and 6 months post-stroke, the ability to at least move any muscle of the hands and feet with grade two on manual muscle test grading, and the ability to understand and follow verbal commands accordingly. Individuals with the following criteria were excluded from the study: those with metal in the cranium, cochlear implants, or cardiac pacemakers; those with unstable epileptic disorders; those with associated comorbid diseases such as cancer, HIV, and hepatitis; and those with any other neuromuscular disorders that affect the subject’s capability to exercise.

### Study outcomes

The following outcome measures were used. The Trunk Impairment Scale (TIS) is a tool to assess trunk performance in post-stroke individuals (Verheyden et al., 2004; Fujiwara et al., 2004; Verheyden & Kersten, 2010). The Fugl-Meyer Assessment of Motor Recovery after stroke in the upper extremity subsection (FMUE) is a reliable and valid scale to assess upper limb motor functions (Hiengkaew, Jitaree & Chaiyawat, 2012; Page, Fulk & Boyne, 2012). The Wolf Motor Function Test (WMFT) is a stroke-specific scale to measure upper extremity activities (Chen et al., 2012) and it has Time Score (WMFT-TS) in seconds and Function Score (WMFT-FS) in grades. The Ten Meter Walk Test (TMWT) assesses walking or gait speed (Collen, Wade & Bradshaw, 1990; Tyson & Connell, 2009). Finally, the Stroke-Specific Quality of Life scale (SSQOL) assesses health-related quality of life in stroke survivors (Muus, Williams & Ringsberg, 2007; Boosman et al., 2010).

### Equipment

Along with all the outcome measures, we used the Sotirex Medical 1X1 tDCS device with its accessories for providing tDCS. For providing PNF and conventional physiotherapy, we used beds, chairs, mats, gym balls, sandbags, benches, parallel bars, step-ups, and stairs.

### Interventions

An independent assessor with a doctorate in physical therapy and 15 years of experience in neurological rehabilitation conducted pre- and post-intervention testing of all the outcome measures and he is blinded for the allocation. After the assessment, another independent assessor with a doctorate in physical therapy and 15 years of experience in neurological rehabilitation provided the participants with treatment. All three groups received rehabilitation sessions lasting 90 min in duration, 4 times per week, for 6 weeks. Each group had a treatment protocol as follows.

Group 1 (CPT) received conventional physical therapy (CPT) alone. The whole person is focused on the CPT interventions which include upper limb, trunk and lower limb. Each patient had undergone detailed neurological physical therapy evaluation with a focus on motor and functional components. Interventions were performed according to the functional limitations and the capability of each patient. The treatment strategies included muscle elongation exercises for tight muscles; strengthening exercises for weak muscles; mat activities such as bridging, rolling and sitting, weight-bearing, and shifting in sitting and standing; range of motion exercises; and gait training activities.

**Figure 2:**
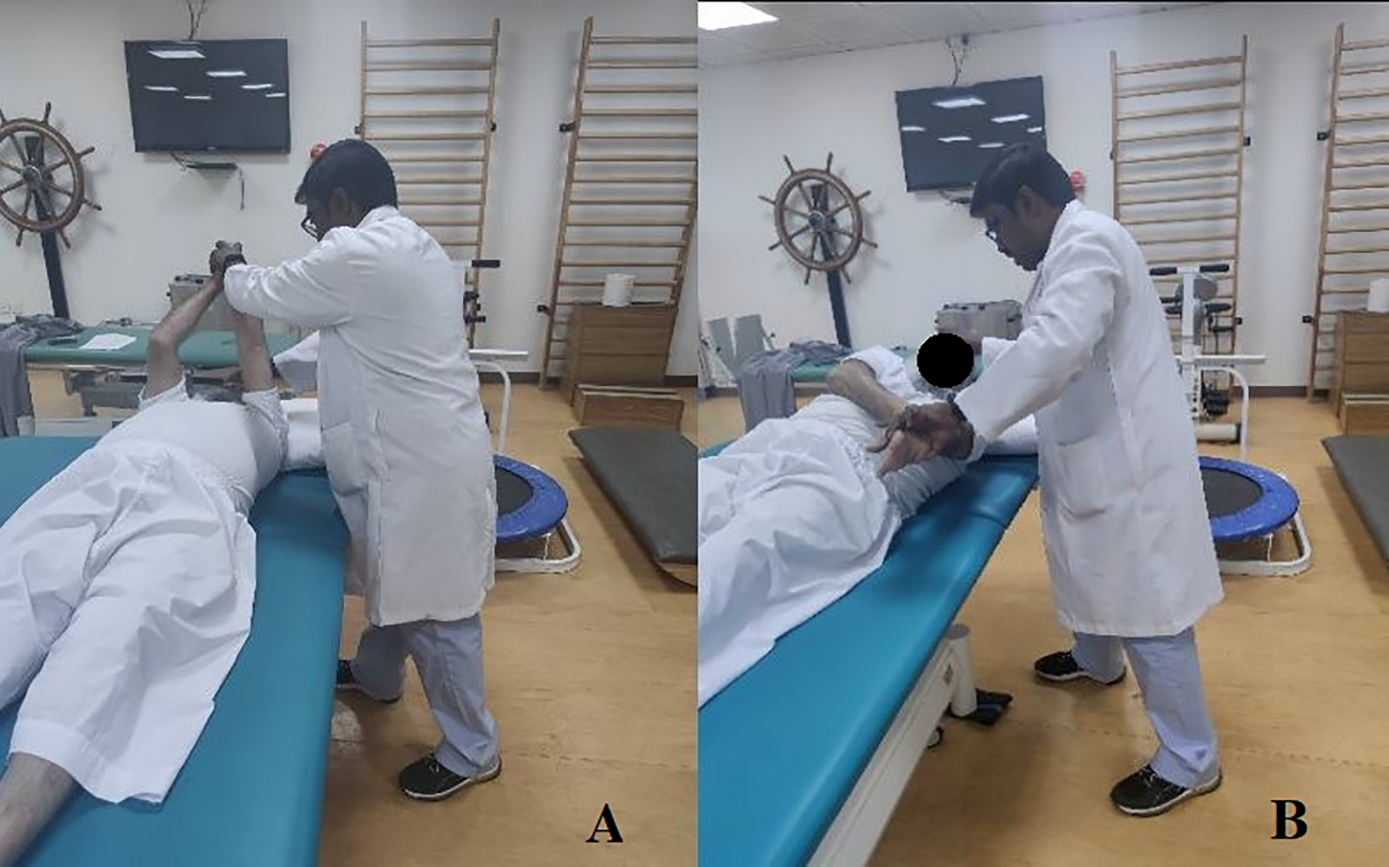
Demonstrating the PNF trunk chopping pattern in supine position with minimal resistance from the therapist. (A) Starting position (B) During the chopping pattern.

Group 2 (StDCS) received CPT, trunk targeted PNF + sham tDCS. It comprised 20 min of sham tDCS followed by 30 min of trunk targeted PNF and then 40 min of CPT. The CPT and PNF exercises were based on functional limitations and the capability of each patient. The therapist was certified in advanced levels of the PNF concept and followed the fundamental principles of the PNF method, such as tactile stimulation, visual and auditive stimulation, traction/approximation, timing, and repetitions. Trunk chopping and lifting patterns, bilateral lower limb/upper limb patterns, combined scapular patterns, and pelvic patterns were used to facilitate trunk. These exercises were done in positions such as supine, side-lying, prone, sitting, and standing based on patient capabilities. PNF techniques were incorporated based on the patient requirements, although stabilizing reversals and rhythmic stabilization were mandatory techniques. For sham tDCS, the stimulation points were C3–C4 of the international EEG 10–20 system. The anode was placed on the affected side and the cathode on the unaffected side. The electrode size was 5 × 5 cm, and saline soaked sponges were used on these electrodes. The intensity of the current used was 2 mA, and the duration of the stimulation was 20 min. The ramp up and down period was 30 s each, but only the difference was that we used the machine’s sham button during the beginning of the tDCS procedure. Figure 2 demonstrates a sample of trunk pattern PNF activity on the patient with stroke.

Group 3 (tDCS) received CPT, PNF, and tDCS. It comprised 20 min of bihemispheric motor cortex tDCS followed by 30 min of trunk targeted PNF and then 40 min of CPT. The stimulation points were C3–C4 of the international EEG 10–20 system. The anode was placed on the affected side and the cathode on the unaffected side. The electrode size was 5 × 5 cm, and saline soaked sponges were used on these electrodes. The intensity of the current used was 2 mA, and the duration of the stimulation was 20 min. The ramp up and down period was 30 s each.

### Statistical analysis

The software language R, version 3.6.3, was used for statistical computing. The level of significance (*p*-value) was kept as <0.05. Descriptive statistics were used for calculating the mean, standard deviation, and percentages for the patient characteristics. The variables were computed for analyzing the normal distribution by using Shapiro–Wilk’s test, and we found that all the variables fell under a normal distribution. A two-way linear mixed model analysis was used to test for main and interaction effects of time (within-subjects factor; levels: pre, post) and group (between-subjects; levels: CPT, StDCS, tDCS) on the study outcomes (TIS, FMUE, WMFT, TMWT, SSQOL). The *post hoc* analysis of inter group differences were also conducted by using Tukey post-hoc tests in cases where the null hypothesis was rejected. An intention-to-treat analysis was performed. Mean differences (MD) with a 95% confidence interval (95% CI) were also calculated.

## Results

All 54 subjects completed their treatment program and were analyzed for the outcome measures. The mean age of our sample was approximately 57 to 58 years old, and the number of days post-stroke was above 60 days. Out of the 54 people included in the study, 29 were males, 25 were females, and most were suffering from ischemic stroke than hemorrhagic strokes. The group’s baseline characteristics regarding age, gender, affected brain side, type of stroke, and the number of days post-stroke are provided in Table 1.

**Table 1:** Patient characteristics expressed in terms of mean ± SD/numbers/frequencies.

Patient characteristics		CPT group	StDCS group	tDCS group
Age (Mean ± SD)		58.78 (5.46)	57.83 (6.72)	58.50 (6.42)
Stroke duration (days; Mean ± SD)		62.78 (20.98)	63.11 (20.97)	62.56 (17.10)
Gender (N.%)	Male	10 (55.56%)	9 (50.00%)	10 (55.56%)
	Female	8 (44.44%)	9 (50.00%)	8 (44.44%)
Affected side (N.%)	Left	10 (55.56%)	10 (55.56%)	9 (50.00%)
	Right	8 (44.44%)	8 (44.44%)	9 (50.00%)
Diagnosis (N.%)	Ischemia	15 (83.33%)	14 (77.78%)	15 (83.33%)
	Hemorrhagic	3 (16.67%)	4 (22.22%)	3 (16.67%)

**Note:**

SD, Standard Deviation; CPT, Conventional Physical Therapy; StDCS, Sham Transcranial Direct Current Stimulation; tDCS, Transcranial Direct Current Stimulation.

**Figure 3:**
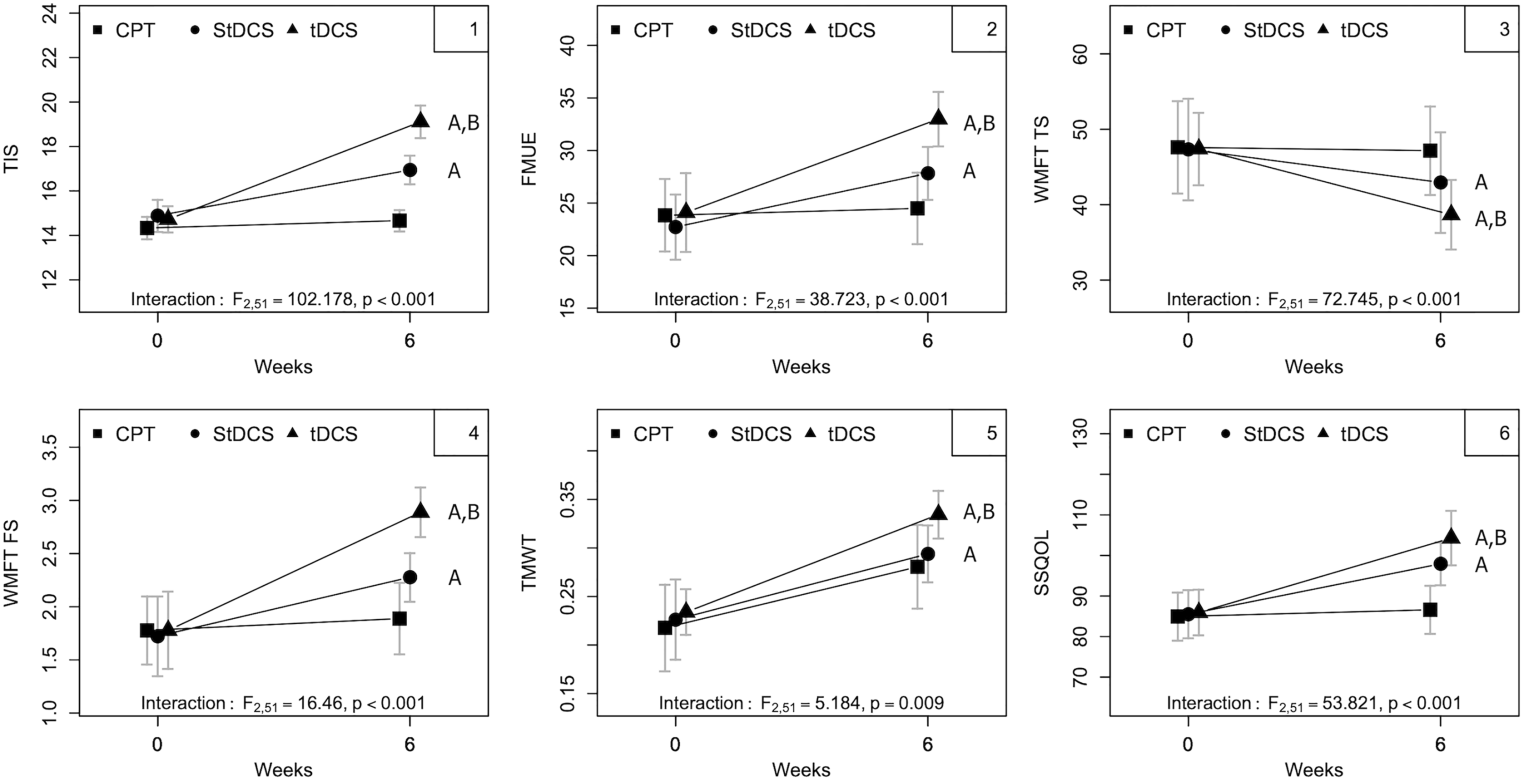
**Showing the interaction effect of time with groups for all the outcome measures.** 1: Trunk Impairment Scale (TIS), 2: FMUE: Fugl-Meyer Upper Extremity Assessment, 3: WMFT (TS): Wolf Motor Function Test Time Score, 4: WMFT (FS): Wolf Motor Function Test Function Score, 5: TMWT: Ten Meter Walk Test, 6: SSQOL: Stroke Specific Quality of Life. CPT: Conventional Physical Therapy, StDCS: Sham transcranial Direct Current Stimulation group, tDCS: transcranial Direct Current Stimulation group. A: Significant compared to CPT, B: Significant compared to StDCS.

The mean and standard deviations of the all the outcome measures pre- and post- intervention among all the three groups is mentioned in Table 2. In the CPT group out of all the outcome measures there is significant improvement seen only in walking speed. However, in the StDCS with PNF, tDCS along with PNF groups had improved significantly in all the outcome measures. The improvements in SSQOL are not significant in CPT group but they were improved significantly in both StDCS and tDCS groups. However, the improvements in tDCS group were superior and significant than the StDCS group in all the outcome measures (*p* < 0.003) (Table 3 and Fig. 3) except in the TMWT. The mean and standard deviations of intra group differences along with level of significance we stated in Table 2.

**Table 2:** Intragroup mean differences for all the patient outcome measures with level of significance.

Outcome measure	Baseline values of the groups			After treatment values of the groups (6 weeks)			Intra group differences from baseline to after treatment		
	CPT Pre Mean (SD) (*n* = 18)	StDCS Pre Mean (SD) (*n* = 18)	tDCS Pre Mean (SD) (*n* = 18)	CPT Post Mean (SD) (*n* = 18)	StDCS Post Mean (SD) (*n* = 18)	tDCS Post Mean (SD) (*n* = 18)	CPT Post-Pre Mean (SD) *p*-value	StDCS Post-Pre Mean (SD) *p*-value	tDCS Post-Pre Mean (SD) *p*-value
TIS	14.33 (1.03)	14.89 (1.45)	14.72 (1.18)	14.67 (0.97)	16.94 (1.30)	19.11 (1.49)	0.33 (0.59) 0.10	2.06 (0.80) <0.001*	4.39 (1.09) <0.001*
FMUE	23.83 (6.94)	22.72 (6.23)	24.11 (7.56)	24.50 (6.87)	27.83 (5.03)	33.00 (5.19)	0.67 (0.49) 0.31	5.11 (3.56) <0.001*	8.89 (3.27) <0.001*
WMFT (TS)	47.61 (12.28)	47.33 (13.54)	47.39 (9.64)	47.17 (11.85)	42.94 (13.39)	38.67 (9.26)	−0.44 (0.92) 0.36	−4.39 (0.92) <0.001*	−8.72 (3.32) <0.001*
WMFT (FS)	1.78 (0.65)	1.72 (0.75)	1.78 (0.73)	1.89 (0.68)	2.28 (0.46)	2.89 (0.47)	0.11 (0.32) 0.37	0.56 (0.51) <0.001*	1.11 (0.68) <0.001*
TMWT	0.22 (0.09)	0.23 (0.08)	0.23 (0.05)	0.28 (0.09)	0.29 (0.06)	0.33 (0.05)	0.06 (0.04) <0.001*	0.07 (0.04) <0.001*	0.10 (0.02) <0.001*
SSQOL	84.94 (11.94)	85.50 (11.97)	85.94 (11.42)	86.61 (11.95)	97.94 (10.73)	104.28 (13.47)	1.67 (0.69) 0.15	12.44 (5.62) <0.001*	18.33 (6.30) <0.001*

**Notes:**

* Significant difference between the comparisons.

SD, Standard Deviation; CPT, Conventional Physical Therapy; StDCS, Sham Transcranial Direct Current Stimulation; tDCS, Transcranial Direct Current Stimulation; TIS, Trunk Impairment Scale; FMUE, Fugl-Meyer Upper Extremity Assessment; WMFT (TS), Wolf Motor Function Test Time Score; WMFT (FS), Wolf Motor Function Test Function Score; TMWT, Ten Meter Walk Test; SSQOL, Stroke Specific Quality of Life.

**Table 3:** Two-way linear mixed model analysis of outcome measures showing the time, group and interaction effects.

Outcome measure	*Post hoc* analysis inter group differences			Time effect		Group effect		Interaction effect	
	CPT-StDCS Post Mean (CI), *p*-value	CPT- tDCS Post Mean (CI), *p*-value	StDCS-tDCS Post Mean (CI), *p*-value	*F*	*p*-value	*F*	*p*-value	*F*	*p*-value
TIS	1.7 [1.24–2.20], <0.001*	4.1 [3.46–4.65], <0.001*	2.3 [1.68–2.98], <0.001*	19.13	<0.001*	377.65	<0.001*	102.17	<0.001*
FMUE	4.4 [2.72–6.17], <0.001*	8.2 [6.64–9.81], <0.001*	3.8 [1.46–6.09], <0.001*	2.42	0.10	163.92	<0.001*	38.72	<0.001*
WMFT (TS)	−3.9 [−4.57 to −3.32], <0.001*	−8.3 [−9.93 to −6.63], <0.001*	−4.3 [−5.98 to −2.68], <0.001*	0.62	0.54	259.91	<0.001*	72.74	<0.001*
WMFT (FS)	0.4 [0.15–0.73], <0.003*	1.0 [0.64–1.36], <0.003*	0.6 [0.15–0.96], <0.003*	3.5	0.04*	69.07	<0.001*	16.46	<0.001*
TMWT	0.1 [−0.02 to 0.03], 0.55	0.4 [0.01–0.06], 0.54	0.3 [0.01–0.06], 0.55	1.21	0.30	219.18	<0.001*	5.18	0.01*
SSQOL	10.8 [8.07–13.49], <0.001*	16.7 [13.6–19.7], <0.001*	5.9 [1.85–9.93], <0.001*	2.94	0.06	264.36	<0.001*	53.82	<0.001*

**Notes:**

* Significant difference between the comparisons.

SD, Standard Deviation; CI, 95% Confidence interval; CPT, Conventional Physical Therapy; StDCS, Sham Transcranial Direct Current Stimulation; tDCS, Transcranial Direct Current Stimulation; TIS, Trunk Impairment Scale; FMUE, Fugl-Meyer Upper Extremity Assessment; WMFT (TS), Wolf Motor Function Test Time Score; WMFT (FS), Wolf Motor Function Test Function Score; TMWT, Ten Meter Walk Test; SSQOL, Stroke Specific Quality of Life.

All outcomes—TIS, FMUE, WMFT TS, WMFT FS, TMWT, SSQOL—showed interaction effects (all *p* < 0.01 or lower). Overall, pre–post mean differences demonstrate a more substantial improvement in the active tDCS group, followed by sham stimulation associated with the PNF group (StDCS) than the group that received CPT alone. Table 3 show the summary of group, time and interaction effects between baseline (pre) to 6 weeks of treatment (post) and of groups as analyzed using a two-way linear mixed model for all five outcome measures.

The main effects of the group were also observed for all outcomes (all *p* < 0.001). Analysis of the 95% CI shows that between-group MD was larger for the CPT-tDCS group than for either StDCS-tDCS or CPT-StDCS in all study outcomes (in absolute values). Further, the between-group MD was larger for StDCS-tDCS than CPT-StDCS for TIS, WMFT TS, WMFT FS, and TMWT. However, for FMUE and SSQOL, in contrast, the absolute between-group MD was larger for CPT-StDCS than StDCS-tDCS (Table 3).

The main effect for time was observed for TIS and WMFT functional scores (*p* < 0.001 and *p* = 0.04, respectively). An overall trend to increase (for TIS, FMUE, WMFT FS, TMWT, and SSQOL) and decrease the MD (for WMFT TS) over time was observed. Analysis of the 95% CI shows that larger MDs (within-group) for TIS and WMFT were observed for tDCS than for the StDCS and CPT groups (Table 3). Fig. 3 shows improvement from baseline to 6 weeks in all the three groups on five outcome measures. No major adverse effects were reported after tDCS sessions. Three patients experienced mild dizziness post-tDCS treatment, but this dizziness subsided within an hour. There was no loss to follow-up.

## Discussion

This randomized, double-blind trial assessed the effects of tDCS neuromodulation combined with a robust clinical approach: trunk targeted PNF on impairments, activity limitations, and participation restrictions of individuals with subacute stroke. In this study, we found that this combination of tDCS with trunk PNF was highly beneficial to improve trunk capacity, upper extremity function, gait speed, and quality of life after stroke. The group receiving bihemispheric tDCS in associated with trunk-targeted PNF along with CPT achieved greater improvements most of the outcome measures than both the group who received sham tDCS associated with trunk-targeted PNF and CPT as well as the group who received CPT alone.

The advantage of PNF over CPT is that it is a facilitation of movements and muscles in a three-dimensional manner, that is, in flexion/extension, abduction/adduction, and rotation planes together. Along with the above, the resistance, timing, and techniques of PNF further stimulate muscle and neuronal interactions and promote recovery in peripheral and central systems (Shim et al., 2020).

tDCS effects over the motor cortex are considered polarity dependent. Many aspects can influence the excitability changes induced by the stimulation, including the strength of the current applied and the duration of the application. In general, anodal tDCS is nevertheless considered to increase motor cortex excitability, whereas cathodal tDCS decreases this excitability (Nitsche & Paulus, 2000). These immediate changes are due to subthreshold changes in membrane polarization. Long-term effects, which persist after stimulation, are more likely to be related to the release of neurotransmitters such as GABA and alteration in NMDA receptor sensitivity. Further, the increase in neuronal connectivity and angiogenesis due to tDCS will enhance brain capacity and motor function (Roche, Geiger & Bussel, 2015).

Unilateral motor cortex tDCS of affected and non-affected sides with anodes and cathodes, respectively, revealed significant enhancement in motor function in the previous literature (Kang, Summers & Cauraugh, 2016). However more recent studies combined affected side anodal and non-affected side cathodal tDCS stimulation through a bilateral tDCS montage aiming to further enhance motor recovery in stroke subjects (Di Lazzaro et al., 2014). Lindenberg et al. (2010) demonstrated that the group of stroke patients receiving bilateral tDCS stimulation associated with physical/occupational therapy showed greater motor function improvement. Even more impressive, these behavioral effects accompanied functional changes in the motor/premotor cortex activation ipsilateral to the lesion during elbow and wrist movement in the group of stroke patients receiving bilateral tDCS stimulation. In contrast, no changes were reported in Lindenberg et al. (2010) sham stimulation control group.

We found superior effects in the tDCS group for the impairments, activity limitations related to the upper and lower libs, and participation restrictions in subjects with subacute stroke. Its success may be related to the rehabilitation protocol chosen. Many previous studies endeavored to incorporate the association of tDCS to enhance the effects of different rehabilitation approaches to improve the motor function of stroke subjects, for example, through employing constraint-induced movement therapy or (Malcolm et al., 2007) physical or occupational therapy using motor task training (Lindenberg et al., 2010). However, this study is the first that combines a PNF approach center in ameliorating trunk control with bihemispheric tDCS. Trunk control is both crucial to good functionality of upper and lower extremities and a key element to be addressed during the rehabilitation of stroke subjects (Karthikbabu et al., 2012; Lee & Kim, 2018). We hypothesize that the increased excitability caused by Anodal tDCS under the affected brain was further enhanced by trunk targeted PNF activities and promoted stronger neuromuscular interactions (Nitsche & Paulus, 2000). Due to continuous training for 6 weeks, there might be more neuronal connectivity leads to structural and functional neuroplastic changes (Roche, Geiger & Bussel, 2015). A combination of the neuromodulation effects of tDCS (Nitsche & Paulus, 2000) along with enhancements in trunk stability, range of motions, coordination, muscle length, and strength occurred due to trunk PNF (Adler, Beckers & Buck, 2014) would have caused the improvements found in the present study.

The functionality changes of the upper limb, trunk, and gait were not only of statistical significance but also better than minimal clinical importance. Lombardi et al. (2017) estimated the minimal detectable change (MDC) for TIS to be 1.44 to 4.55. In our study, the two groups enrolled in trunk PNF exercises achieved this range of MDC but not the control group. Similarly, Pandian & Arya (2014) also proposed MDC and MCID for the FMUE scale in stroke subjects as a 3.5 difference and a 5.7 difference in scores, respectively. In our study, all three groups had achieved MDC, but only in tDCS with the trunk PNF exercise did the group meet the MCID values (Table 2). This result indicates that combining trunk PNF with tDCS was more beneficial for upper extremity functions.

The MDC and CID for the WMFT performance time were 4.36 s and 1.5–2 s, respectively. The MDC and CID for WMFT functional ability scale were 0.37 and 0.2–0.4 points, respectively. In our research, the control, sham tDCS, and tDCS groups had changes in mean ± standard deviation of performance time of 0.44 ± 0.92, 4.39 ± 0.92, 8.72 ± 3.32 scores, and the functional ability scale was 0.11 ± 0.32, 0.56 ± 0.51, and 1.11 ± 0.68, respectively. Of all three groups, the control group did not reach the MDC or CID for both performance time and functional ability scale, while the sham tDCS and tDCS group achieved MDC and CID values. However, only in tDCS with the trunk PNF exercise group had achieved both higher values of MDC and CID than the sham tDCS group (Table 2). This indicates that coalescing PNF with tDCS was more beneficial in clinical improvements of upper extremity functions (Lin et al., 2009).

Perera et al. (2006) determined small meaningful change for TMWT as 0.05 m/s and substantial meaningful change as 0.10 m/s. In our study, the control, sham tDCS and tDCS groups pre- to post-differences were 0.06 ± 0.04, 0.07 ± 0.04, and 0.10 ± 0.02 m/s. All three groups achieved small meaningful change, but only combining trunk-targeted PNF with tDCS had achieved significant meaningful change. This result indicated that combining these two methods along with CPT can make a substantial change in the gait speed of individuals with stroke.

Cho & Cha (2014) investigated the effect of combining the trunk PNF exercises with tDCS over the lower extremity function among 31 patients with stroke (15 in the tDCS group, and 16 in the control group). Eighteen sessions of treatment in 6 weeks’ time showed a significant positive effect over walking speed in both groups, but there was no difference between groups (Cho & Cha, 2014). In our study, there was interaction between groups and time and a significant difference between experimental groups for the 10-Meter Walk Test, with a larger pre–post mean difference for the tDCS group. The discrepancy of the results can, at least in part, be attributed to treatment duration and frequency, both of which are higher in the present study. Another critical difference between the protocols of both studies is the electrode montage.

Comparing our results with similar studies and MCD and CID demonstrates that bihemispheric tDCS, along with trunk PNF and CPT, is a solid combination to improve stroke patient capabilities. We could not do any MRI or EEG related outcomes, future studies employing neuroimaging and electrophysiological outcomes should investigate the brain structural and functional changes. Since we studied these beneficial effects of PNF and tDCS on supratentorial strokes after an average of 2 months after the stroke, the applicability of study findings on all stroke populations is queried. Hence, the influence of including the variety of stroke lesion (cortical or subcortical) and time since stroke over the treatment responsiveness, however, remain to be determined. Finally, multicenter clinical trials with follow-up evaluations and including independent PNF groups are encouraged to confirm the clinical efficacy of bihemispheric-tDCS combined with trunk-targeted PNF.

## Conclusion

Six weeks of trunk-targeted PNF, combined with bihemispheric tDCS and CPT, can improve trunk capacity, upper extremity function, gait speed, and quality of life after stroke.

## Supplemental Information

10.7717/peerj.13329/supp-1 Supplemental Information 1 Clinical Trial Protocol.

10.7717/peerj.13329/supp-2 Supplemental Information 2 Consort check list.

10.7717/peerj.13329/supp-3 Supplemental Information 3 Raw data.

## Additional Information and Declarations

### Data Availability

The following information was supplied regarding data availability: The raw data is available in the Supplemental File.

### Clinical Trial Registration

The following information was supplied regarding Clinical Trial registration: CTRI/2019/09/021270.
