# Supplementary material for: Transcranial direct current stimulation combined with trunk-targeted, proprioceptive neuromuscular facilitation in subacute stroke: a randomized controlled trial
Source: PeerJ. 2022 Apr 28;10:e13329. doi: 10.7717/peerj.13329 (PMC9057289; doi:10.7717/peerj.13329)
Supplement: Supplemental Information 1 [file peerj-10-13329-s001.pdf]

**FULL DETAILS (Read-only)**

| | |
|---|---|
| **CTRI Number** | **CTRI/2019/09/021270** [Registered on: 17/09/2019] **Trial Registered Prospectively** |
| **Last Modified On:** | 14/09/2019 |
| **Post Graduate Thesis** | No |
| **Type of Trial** | Interventional |
| **Type of Study** | Physiotherapy (Not Including YOGA) |
| **Study Design** | Randomized, Parallel Group, Active Controlled Trial |
| **Public Title of Study** | Electrical brain stimulation along with physical therapy effect on improving function in patients with sub-acute stroke |
| **Scientific Title of Study** | Transcranial direct current stimulation combined with trunk targeted proprioceptive neuromuscular facilitation and its effects on impairments, activity limitations and participation restrictions of subjects with sub-acute stroke: A randomized controlled trial |

**Secondary IDs if Any**

| Secondary ID | Registry |
|---|---|
| NIL | NIL |

**Details of Principal Investigator or overall Trial Coordinator (multi-center study)**
Modification(s)

| | |
|---|---|
| **Name** | Ajay Kumar Midde |
| **Address** | Department of Physiotherapy, Krishna Institute of medical Sciences, Ministers road, Secunderabad<br><br>Hyderabad<br>TELANGANA<br>500003<br>India |
| **Phone** | 00919908167776 |
| **Fax** | |
| **Email** | ajaymidde85@gmail.com |

**Details Contact Person Scientific Query**
Modification(s)

| | |
|---|---|
| Name | Ajay Kumar Midde |
| **Address** | Krishna Institute of medical Sciences, Ministers road, Secunderabad<br><br>Hyderabad<br>TELANGANA<br>500003<br>India |
| **Phone** | 00919908167776 |
| **Fax** | |
| **Email** | ajaymidde85@gmail.com |

**Details Contact Person Public Query**
Modification(s)

| | |
|---|---|
| **Name** | Ajay Kumar Midde |
| **Address** | Krishna Institute of medical Sciences, Ministers road, Secunderabad<br><br>Hyderabad<br>TELANGANA |

| | |
|---|---|
| | 500003<br>India |
| **Phone** | 00919908167776 |
| **Fax** | |
| **Email** | ajaymidde85@gmail.com |

| | |
|---|---|
| **Source of Monetary or Material Support** Modification(s) | Department of Medical rehabilitation Sciences, College of Applied Medical Science, C/3/108, Guraiger, King Khalid University, Abha, 61421, Kingdom of Saudi Arabia |
| **Primary Sponsor** | **Name**: King Khalid University<br>**Address**: C/3/108, Building C, Guraiger, King Khalid University, Abha, Kingdom of Saudi Arabia<br>**Type of Sponsor**: Government medical college |
| **Details of Secondary Sponsor** | **Name**: NIL<br>**Address**: NIL |
| **Countries of Recruitment** | India<br>Saudi Arabia |

**Sites of Study** Modification(s)

No of Sites = 1

| Contact Person | Name of Site | Site Address | Phone/Fax/Email |
|---|---|---|---|
| Dr Jaya Shanker Tedla | Krishna Institute of Medical Sciences | Department of Physiotherapy, 2nd floor, New Building, Ministers Road, Secunderabad Hyderabad | 7674897379<br>jtedla@kku.edu.sa |

**Details of Ethics Committee**

No of Ethics Committees= 1

| Name of Committee | Approval Status |
|---|---|
| Ethics Committee for Scientific Research | Approved |

**Regulatory Clearance Status from DCGI**

| Status |
|---|
| Not Applicable |

**Health Condition / Problems Studied**

| Health Type | Condition |
|---|---|
| Patients | Cerebral infarction, unspecified |

**Intervention / Comparator Agent**

| Type | Name | Details |
|---|---|---|
| Comparator Agent | Conventional Physical Therapy | In this group, the total intervention will be for one and a half hour. Muscle elongation exercises for tight muscles, strengthening exercises for weak muscles, weight-bearing and shifting in sitting and standing, |

| | | | |
|---|---|---|---|
| | | | range of motion exercises and gait training activities. |
| | Intervention | Conventional Physical Therapy, Trunk targeted proprioceptive neuromuscular facilitation (PNF) | In this group, the total intervention will be for one and half hour. It includes one hour of Conventional Physical Therapy and 30 minutes trunk targeted PNF. The PNF exercises were based on functional limitation and capability of an individual patient. The therapist will strictly follow the fundamental principles of the PNF method like manual contact, body position and body mechanics, verbal commands, vision, traction/approximation, stretch, timing, and repetitions. Trunk chopping and lifting patterns, bilateral lower limb/upper limb patterns, combined scapular, and pelvic patterns will be used to facilitate trunk. These exercises will be done in different positions like supine, side-lying, prone, sitting and standing based on patient capabilities. Techniques of PNF are incorporated based on patient requirement but Stabilizing reversals / Rhythmic stabilization are mandatory techniques. |
| | Intervention | Trans Cranial Direct Current Conventional Physical therapy , Stimulation with Trunk targeted and Proprioceptive Neuro muscular facilitation | In this group, the total intervention will be for one and a half hour. It includes 40 minutes of Conventional Physical Therapy, 30 minutes trunk targeted PNF and 20 minutes tDCS. Details of tDCS are provided below. tDCS treatment parameters Machine used Neuro-Conn DC stimulator plus or Sotirex tDCS machine Montage basis International EEG 10 – 20 system Software used to test montage Bonsai—Model Solution Analyzer Montage used Bicephalic Montage Anode on affected side C3-C4 Cathode on unaffected side C3-C4 Pad size 5X5 cm The intensity of the current 2 mA Duration of treatment 20 min Number of sessions per week 4 Total number of treatment weeks 6 Total number of session 24 |
| **Inclusion Criteria** | **Age From** | 18.00 Year(s) | |
| | **Age To** | 75.00 Year(s) | |
| | **Gender** | Both | |
| | **Details** | 1. Age range 18-75 years<br>2. First-time supratentorial stroke diagnosed by MRI/CT scan<br>3. Between 7 days to 6 months post-stroke<br>4. Able to understand and follow the commands | |
| **ExclusionCriteria** | **Details** | 1. Any metals in the cranium/ cochlear implants/ cardiac pacemakers<br>2. Having an unstable epileptic disorder<br>3. Associated comorbid diseases like cancer/ HIV/Hepatitis or any other neuromuscular disorders which affect the subjects capability to do exercise | |
| **Method of Generating Random Sequence** | Stratified block randomization | | |
| **Method of** | Sequentially numbered, sealed, opaque envelopes | | |

| | |
|---|---|
| **Concealment** | |
| **Blinding/Masking** | Participant, Investigator and Outcome Assessor Blinded |
| **Primary Outcome** | <table><tr><th>Outcome</th><th>TimePoints</th></tr><tr><td>1. Trunk impairment scale measures trunk activity and measured as a clinical test 2. Fugl-Meyer Assessment of Motor Recovery after stroke (FMA) measures upper and lower limb activity and measured as a clinical test 3. 10 Meter Walk Test (10mWT) measures gait speed and measured as a clinical test 4. Timed up and go test measures balance and gait activity<br>5. Wolf Motor Function Test measures upper limb activity and measured as a clinical test<br>6. Stroke Specific Quality of life</td><td>Baseline and after 6 weeks</td></tr></table> |
| **Secondary Outcome** Modification(s) | <table><tr><th>Outcome</th><th>TimePoints</th></tr><tr><td>1. Age 2. Gender 3. Stroke Onset 4. Type of Stroke 5. Severity of Stroke 6. Type of Treatme</td><td>These secondary outcomes required only for doing correlations they are obtained on week one.</td></tr></table> |
| **Target Sample Size** Modification(s) | **Total Sample Size=**"54"<br>**Sample Size from India=**"27" |
| **Phase of Trial** | Phase 1/ Phase 2 |
| **Date of First Enrollment (India)** | 17/09/2019 |
| **Date of First Enrollment (Global)** | 17/09/2019 |
| **Estimated Duration of Trial** | **Years=**"0"<br>**Months=**"10"<br>**Days=**"0" |
| **Recruitment Status of Trial (Global)** Modification(s) | Not Yet Recruiting |
| **Recruitment Status of Trial (India)** | Not Yet Recruiting |
| **Publication Details** Modification(s) | Nil |
| **Brief Summary** | Background and study aim: Stroke is the foremost cause of death and disability throughout the world. Combining the neuromodulation capability of Transcranial Direct Current Stimulation (tDCS) with a strong clinical treatment tool like Proprioceptive Neuromuscular Facilitation (PNF) could make excellent improvements in the lives of stroke population. tDCS is a non-invasive, painless brain stimulation treatment that uses direct electrical currents to stimulate specific parts of the brain. A constant, low-intensity current is passed through two electrodes placed over the head which modulates brain cell activity. PNF stretching is an advanced form of flexibility training. It involves the contraction and stretching of muscles. Currently, there is dearth of literature pertaining to this combination of |

| | |
|---|---|
| | tDCS with PNF in improving function and quality of life in subjects with subacute stroke.<br>The aim of this study is to find the effect of tDCS combined with trunk targeted PNF on impairments, activity limitations and participation restrictions of subjects with sub-acute stroke<br>Who can participate? Stroke patients aged between 18-75 years who meet the inclusion criteria<br>What does the study involve? The participants undergo some evaluations, tDCS, and physical exercise treatments over a six week period.<br>What are the possible benefits and risks of participating? There is a possibility that the patient functional capacity will improve and there is no critical risk of participating in the study Some subjects can feel some fatigue after the exercise<br>Where is the study run from? King Khalid University, Saudi Arabia<br>When is the study starting and how long is it expected to run for? May 2019 to May 2020<br>Who is funding the study? Investigator-initiated and funded<br>Who is the main contact? Dr. Jaya Shanker Tedla jtedla@kku.edu.sa |

Close
